# Conceptions of Healthy Aging Held by Relatives of Older Persons in Isan-Thai Culture: A Phenomenographic Study

**DOI:** 10.1155/2018/3734645

**Published:** 2018-01-14

**Authors:** Pornpun Manasatchakun, Åsa Roxberg, Margareta Asp

**Affiliations:** ^1^Boromarajonani College of Nursing, Udon Thani, Thailand; ^2^School of Health and Welfare, Halmstad University, Halmstad, Sweden; ^3^VID Specialized University, Bergen, Norway; ^4^School of Health, Care and Social Welfare, Mälardalen University, Eskilstuna, Västerås, Sweden

## Abstract

In Thailand, family nurses are expected to provide support for older persons and their family members to promote healthy aging. Family bonds are strong, and relatives are expected to take care of their older family members. However, there is limited research on how older persons' family members perceive healthy aging. This study aimed to describe the conceptions of healthy aging held by the children and grandchildren of older persons in northeast Thailand. In a phenomenographic study, 14 interviews were performed to qualitatively analyze different conceptions of healthy aging. Four descriptive categories emerged: being independent, not being afflicted by diseases or illnesses, being a giver and a receiver, and being wise. The conceptions of healthy aging entail both autonomy and interdependence. The relative's perspective needs to be considered when policies relating to healthy aging are implemented in the community and when family nurses provide support to families to promote healthy aging.

## 1. Introduction

Thailand's healthcare system emphasizes the importance of family participation in care for older persons [1]. A family nurse is the key person who works with older clients and their family members to provide care and support those people's health. In Thailand, a family nurse is defined as a person who takes responsibility for public health, holistic healthcare, and enhancing the quality of life among the population [2]. The roles of a family nurse include not only identifying health problems and managing chronic and acute medical conditions but also collaborating with families and providing health services [2]. These tasks encompass preventing diseases and promoting health for families [2]. Family nurses have traditionally been expected to provide support for older persons and their family members. Family members play a key role as the primary caregivers of older persons in Thai society, especially in the northeastern region of Thailand, also known as Isan. Thus, family nurses should value the role of family when engaging older persons in their care. With a growing aging population, the Thai government has developed health policies to maintain the older population's health and improve their quality of life [3]. The promotion of healthy aging is one of the key strategies adopted in the national agenda for meeting the challenges of caring for the older population [4].

The concept of healthy aging is adopted and studied throughout the world [5, 6]. In Thailand, studies of healthy aging have focused on older persons' perspectives [7–9]. The literature indicates that healthy aging is facilitated not only by older persons themselves but also by their families and culture [6, 10]. To promote healthy aging and encourage family members to work together with healthcare providers, family nurses must consider the relatives' perspective, as relatives contribute to the improvement of health among older persons. Understanding what healthy aging means to family members of older persons may be helpful when collaborating with them and their families in the promotion of health among people in the northeast of Thailand.

### 1.1. Healthy Aging: Positive Outcomes of Old Age and Policies for Responding to the Aging Situation in Thailand

There are many terms that can be used to explain the maintenance of well-being in older persons [6], such as successful aging [11], active aging [12], and healthy aging [6]. Healthy aging is a concept that covers the multidimensional aspects of older persons' lives and may help incorporate the overlapping concepts of active aging and successful aging [6]. Moreover, healthy aging is viewed as a complex concept that involves multiple dimensions and relates to physical, mental, and social well-being [8]. Previous studies have shown that healthy aging entails achieving positive health outcomes, maintaining well-being throughout one's life, and preventing long-term health conditions [6, 10].

Healthy aging has become a major consideration for global policy-makers aiming to preserve the health and improve the quality of life of the older population [13]. As in many countries worldwide, Thailand is currently facing the problem of an aging population [14]. Older persons, their families, and the government need to be aware of the increased risks of chronic diseases and disabilities associated with old age [15]. The Thai government is concerned about the aging of the Thai population. Therefore, healthy aging is one of the goals of the national plan for older persons, which aims to provide support for older individuals to enable them to maintain a healthy lifestyle and to reduce long-term care costs [3]. Implementation of this plan challenges family nurses to achieve the goal of promoting healthy aging.

### 1.2. Family: The Unit Responsible for the Informal Care of Older Persons in Northeast Thailand

Family is viewed as a unit of care [16] and represents the context for individual development, according to one dimension of family nurses [17]. The family has the primary responsibility for advocating the development of family members. The family serves as the context for a client's health, which means that the focus is on the client, and the family is viewed in the background of the client [17]. The individual's health influences the family, and the capability of the family has an impact on the health of individual members [17]. When caring for older persons and promoting their health, the family nurse should be concerned with both the older person and the family members.

In Thai society, family is highly valued [2]. The northeast of Thailand, also referred to as Isan, has a large population of older persons [18] and is afflicted by poverty, especially among the older population [19]. The children of the current generation of older persons remain an important support group in the northeast of Thailand [20, 21]. This importance may stem from the Isan culture's long tradition of paying respect to older persons and the cultural belief in the concept of repaying one's parents [20]. According to Isan cultural values, children are obliged to care for their parents based on Buddhist customs [20–23]. Children and other family members serve as informal caregivers who play an important role in supporting their parents both socially and financially [21]. Older persons still receive support from their family members (e.g., daughters, sons, and grandchildren) to fulfill their needs [21]. However, societal changes in Isan are affecting families and older persons' lives. In Isan, similar to other regions in Thailand, the family structure has shifted from the extended family to the nuclear family. Older persons and young persons have become separated. Young adults tend to move to larger cities featuring centers of commerce to seek economic opportunities [24, 25]. Some adults in Isan leave their own children with their parents and emigrate to the capital city or to other countries to work [21]. A previous study [26] showed that, compared with earlier generations, the current younger generation in the Isan region has fewer opportunities to stay with their older relatives. Sudnongbua et al. [26] reported that older persons in the Isan region felt abandoned because their children had migrated to other areas to work. The consequence of social change is a challenge for family nurses and provides an opportunity for family nurses to improve health among older persons and support their families who live in the northeast of Thailand.

### 1.3. Family Members of Older Persons and Healthy Aging in Northeast Thailand

A previous study of healthy aging in the Isan region showed that older persons' perspectives on healthy aging are related to the support that they receive from family members [7]. However, no studies focusing on the conceptions of healthy aging held by the relatives of older persons in the Isan region have been conducted. The understanding of healthy aging that is held by the relatives of older persons in Isan-Thai culture needs to be expanded. Such studies may enable family nurses to broaden their views regarding the meaning of healthy aging and provide a foundation for developing care practices consistent with healthcare policies. Therefore, the aim of this study was to describe the conceptions of healthy aging held by children and grandchildren caring for older persons in northeast Thailand.

## 2. Methodology

### 2.1. Study Design

This study has a descriptive and phenomenographic design with an epistemological basis in lifeworld theory, as suggested by Ashworth and Lucas [27, 28]. This methodology was used because phenomenography and lifeworld theory aim to explain how human beings derive meaning from their connections with the world around them [29, 30]. One's lifeworld is the everyday life that he or she experiences [29, 31], and each individual experiences things in his or her lifeworld as different phenomena [29]. Phenomenography uncovers different qualitative conceptions of specific phenomena [30, 32]. The phenomenon of interest in this study was the notion of healthy aging.

### 2.2. Study Setting and Participants

This study was set in Udon Thani Province, which is located in northeast Thailand and features a growing population of people aged 60 years or older. We selected this province as the setting because it exhibits both rural and urban characteristics and is an example of an area undergoing modernization and growth [33]. A purposive sampling technique was used to select the participants from a list of family members caring for 17 older persons from 17 families who participated in a previous study on healthy aging in the northeast region of Thailand [7]. Various background characteristics, such as age, sex, marital status, and educational level, were used as inclusion criteria. To ensure that the conceptions of healthy aging were not drawn from older persons, this study included older persons' children and grandchildren aged < 60 years. Three family members were excluded because they were the spouses of the participants in a previous study on healthy aging who were aged > 60 years.

Twelve children and two grandchildren of older persons (11 women and three men) participated in this study, and the age range varied from 16 to 56 years. Five participants had an elementary school education, six had a high school education, one had a bachelor's degree, and two had a master's degree. Eight participants were married, and six participants were single. All participants were Buddhists. The participants' characteristics are described in Table 1.

### 2.3. Data Collection

The first author collected all data from September to October 2013 using semistructured, in-depth interviews and recorded the interviews to produce verbatim transcripts of each conversation. All the participants agreed to have their conversations recorded. The interviews were initiated using open-ended questions, such as “What is meant by healthy aging, in your opinion?,” “Could you tell me about healthy aging?,” and “What makes older persons healthy?” Then, the first author asked specific follow-up questions to prompt the participants to reflect on the meaning of the phrase “healthy aging” and on their own thoughts, such as “What do you mean when you say …?” or “Please give me an example of ….” The lengths of the interviews varied, depending on the participants' responses, between 45 and 90 minutes. Each interview was transcribed verbatim, and each transcript was then translated from Thai to English by a professional Thai-English translator to ensure that the translations were accurate [34].

### 2.4. Data Analysis

The authors analyzed the transcribed interviews according to the steps suggested by Dahlgren and Fallsberg [35]. First, in the familiarization step, the first author read the transcriptions several times in both Thai and English to gain an overview of the relatives' conceptions of healthy aging. This step was necessary to verify the accuracy of the transcriptions in Thai and English. The first author confirmed that the transcriptions completely retained the meaning of the responses. The English transcripts were then used throughout the analysis process. The other authors read the English transcripts several times to familiarize themselves with the content. In the second step, the meaning units associated with healthy aging were condensed into short statements. In the third step, called the comparison step, similarities and differences in the dialogue excerpts were identified. Then, similar statements were grouped into descriptive categories in the fourth step. In the fifth step, or the articulating step, embedded conceptions in each group of meaning units were identified. The sixth step was the labeling step, in which the authors named the various categories. The final step was the contrasting step, in which the differences and similarities between categories were considered.

### 2.5. Ethical Approval

We followed the guidelines of the principles outlined in the Declaration of Helsinki [36], which emphasize showing respect for all participants and protecting their rights. The first author orally informed the participants about the study and then provided them with written information detailing the purpose of the study and its possible benefits. The participants could choose the time and place of the interview and were allowed to withdraw from the study at any time. The participants were also ensured that their information would remain confidential. Permission to conduct this study was granted by the Udon Thani Provincial Public Health Office Committee Board in Thailand and the Ethical Review Board of Research Involving Humans. This study was registered with the Research Ethical Review Board of Uppsala, Sweden (Dnr 2013/019).

## 3. Results

The participants' conceptions of healthy aging consisted of four qualitatively different descriptive categories: being independent, not being afflicted by diseases or illnesses, being a giver and a receiver, and being wise. Each descriptive category is presented and described in detail below.

### 3.1. Being Independent

Healthy aging means having a desire to do everything by oneself and to avoid relinquishing control or seeking assistance from family members. This category comprised the following two subcategories: “performing activities without the assistance of others” and “not being a burden to others.”

#### 3.1.1. Performing Activities without the Assistance of Others

Healthy aging means being able to care for oneself, to be free, and to participate in activities considered appropriate for one's age without needing assistance from relatives. Healthy aging also means having the ability to work after retirement to earn an adequate income as a means of supporting oneself financially. One participant described healthy aging as follows:

*My mother is expected to do things by herself. Older persons should work and earn their own income. They can choose what they want to do. Some choose to eat vegetables or fish. Some like chili paste. You just live on your own.* (6)

Healthy aging means having the ability to adjust to different circumstances and having autonomy, which fosters a sense of freedom that prevents individuals from being forced to perform unwanted activities.

#### 3.1.2. Not Being a Burden to Others

According to the participants, healthy aging means not being dependent on family members. Specifically, healthy aging means that one's needs are sufficiently met such that he or she demands nothing. Thus, one who ages healthily lives a sufficient life and does not strive for unnecessary things, offend anyone, or interfere in others' business. One participant described this idea as follows:

*She does what she can and doesn't impose a burden on her children and grandchildren. Someone always expresses concern that our family spoils her. But my mother isn't like that. We don't have to worry about her.* (2)

This statement indicates that healthy aging means not being a burden to others and that one should not impose oneself on one's children or grandchildren.

### 3.2. Not Being Afflicted by Diseases or Illnesses

Healthy aging was described as a lack of degeneration of the body and mind. This concept means that one does not suffer or worry about living with chronic diseases or illnesses. In this state, one has the ability to maintain the functions of his or her body and mind. Healthy aging means having a considerably strong, age-appropriate body and mind, the strength of which is visually apparent to oneself and to others. This category encompassed the subcategories “living with diseases or illnesses” and “exhibiting physical and mental strength.”

#### 3.2.1. Living with Diseases or Illnesses

Healthy aging means having the ability to live with serious diseases or illnesses and to continue performing daily life activities. One relative noted the following:

*My mother is free from illness. Older persons can have some diseases, but not many. Although they have diseases, they effectively live with them. As long as they can simply live according to their routine, they are fine.* (12)

The participants stated that even individuals who have some chronic diseases can exhibit healthy aging, although these individuals must find a way to live with their health conditions and thus live an ordinary life.

#### 3.2.2. Exhibiting Physical and Mental Strength

Healthy aging means being in good physical and mental conditions and free of diseases or illnesses that can limit one's ability to perform daily life activities. Healthy aging also means having a good memory, which is particularly desirable among older persons. Such individuals are able to manage their emotions and are as strong as others of the same age.

*They must be healthy, both physically and mentally, and free of diseases or illnesses. If you have a strong body, you also have a good mind. If you are well on the inside, your physical body is also well. If you are healthy physically, everything is healthy. When you have a positive state of psychological health, everything is fine. If you are calm people around you feel cool.* (9)

Healthy aging means being strong and being a psychologically healthy person. Healthy individuals' bodily functions are clearly connected to their mental functions, linking their inner and outer states.

### 3.3. Being a Giver and a Receiver

The participants conceived of healthy aging as giving and receiving help from others and stated that happiness and satisfaction derive from these exchanges. Furthermore, healthy aging means being generous and performing good deeds in accordance with Buddhist beliefs, resulting in satisfaction, and delight from Buddhist practices. This category comprised the following two subcategories: “giving assistance and receiving support” and “devoting oneself to and being delighted by Buddhist practices.”

#### 3.3.1. Giving Assistance and Receiving Support

Healthy aging means having family members available to provide help, as well as helping children and others by acting as a giver. In this context, healthy individuals are good parents who have raised and provided for their family members but who also receive help from their children and grandchildren.

*She makes bamboo sticks for grilled sticky rice for her son. He sells grilled sticky rice in town, and she helps him. She is happy to do it. I help take care of her bed and her mosquito net. My aunt sells grilled eggs in town. She always buys delicious food for my grandmother when she visits.* (4)

In this context, healthy aging means providing cooking materials for family members and receiving support from family members. It means asking for help, for example, to meet one's daily needs. Healthy aging also means helping family members in their work to support them.

#### 3.3.2. Devoting Oneself to and Being Delighted by Buddhist Practices

Healthy aging means devoting oneself to the beliefs of Buddhism and attaining peace as a result of Buddhist practices. Healthy individuals live virtuous and positive lives, and perform noble deeds. In return, these individuals experience happy lives and good health. Dedicating oneself to Buddhism involves making merit and practicing the Buddhist doctrine.

*She likes to go to temple. The temple is her society. Sometimes, she cuts the grass for the abbot and the monks. The monks support her, and my mother supports them. She offers to make merit in religious ceremonies. My mother lives in the community and adheres to Dharmic principles. Making merit is a sign that one's mind is peaceful and that one's health is also good.* (9)

Healthy aging means that one is dedicated to the act of making merit in accordance with Buddhist teachings. Making merit entails not only doing something good for others but also receiving merit in return, such as happiness, positive energy, peace, and enjoyment.

### 3.4. Being Wise

Healthy aging means being wise, which entails understanding one's own life and life situations that occur. In particular, being wise means having the inner strength to address life's challenges and the knowledge to prevent diseases and take care of oneself. This category consisted of the following two subcategories: “experiencing life as a journey” and “preparing for old age.”

#### 3.4.1. Experiencing Life as a Journey

Healthy aging means having sufficient knowledge—although not necessarily educational knowledge or an educational degree—to address problems in life. In the context of healthy aging, this subcategory refers to life experiences, which are derived from one's lifestyle. Healthy individuals regularly engage in positive practices in their everyday life to gain experiences that can help them make necessary decisions.

*She understands what she needs to do because her life experience helps her. She learns from what she does. If it is good for her, she will continue to do it. She learns from her behavior. My mother has lived long enough to have experienced good and bad events in her life.* (12)

The participants stated that healthy aging means having the ability to learn through life experiences and to experience life as a journey.

#### 3.4.2. Preparing for Aging

Healthy aging means preparing oneself for growing old, which includes being satisfied with one's present life and focusing on the moment. Preparing for aging involves practicing what is beneficial for one's health to continue feeling young.

*People should prepare for what they have to confront when they get old. They have prepared themselves since they were young, that is, our age. My mother has been doing this. She does aerobic dance and jumps rope, for example.* (10)

Healthy aging is conceived of as preparing oneself to face the process of growing old. Preparing for aging includes preventing a decline in functions, for instance, by exercising and maintaining proper nutrition and hygiene.

## 4. Discussion

This study is the first to examine how the children and grandchildren of older persons in northeast Thailand conceive of healthy aging. The participants determined the meaning of healthy aging based on their lived experience of connecting to older persons they lived with and took care of in everyday life. The meaning of healthy aging consists of four descriptive categories: “being independent,” “not being afflicted by diseases or illnesses,” “being a giver and a receiver,” and “being wise.” The findings of this study reveal existential aspects of healthy aging. Additionally, the current study elucidates different conceptions of healthy aging that family nurses should consider when working together with the family members of older persons.

The family is viewed as the context, according to the focus of the family nursing perspective [17], which means that the older person is in the foreground and the family is in the background. Although the older person is the main focus of the promotion of healthy aging, family nurses should also consider the perspectives of older persons' family members. The family is an important unit since the family supports and provides for the older family member. Family nurses therefore need to integrate the conceptions of healthy aging held by the family members of older persons in their care. This does not mean changing their way of thinking but involves being aware that there is not only one conception of healthy aging when participating with the family members of older persons. To work with the family members of older persons, family nurses should respect and try to understand their conceptions of healthy aging. The challenge for family nurses and policy-makers is to find strategies to handle the different ways in which the relatives experience healthy aging. It is obvious that the family members of older persons conceive of healthy aging differently. As the results show, family nurses should equally emphasize the importance of being autonomous and depending on others. Moreover, healthy aging is related to disease and illness but is also focused on wisdom in old age. Therefore, family nurses should apply these perceptions when promoting healthy aging. Another challenge is to integrate the differences between the perspectives of older persons and family members when working with these groups of people. This practice may be useful for supporting good relationships among older persons, family members, and healthcare providers when implementing a plan to promote healthy aging.

A comparison of healthy aging from the perspectives of the children and grandchildren of older persons reveals similarities between healthy aging, successful aging, and active aging. These similarities demonstrate that the meaning of healthy aging relates to the ability to remain active and to maintain the overall functioning. This is in line with the definition of successful aging, which emphasizes healthy cognitive function and focuses on the state of functioning that makes an active life possible [11, 37]. When comparing the findings of this study with the concept of active aging, we found that both healthy aging and active aging refer to the ability to maintain autonomy as well as the importance of independence for the older person [12]. There is little difference between the concepts of healthy aging found in this study and a previous study [38]. That previous study on healthy aging also showed that healthy aging was defined as freedom from chronic illness and impairment [38]. However, in the present study, healthy aging is defined not only as the absence of disease or illness but also the importance of the ability to live with disease or illness.

Our results indicate the diversity of meanings of healthy aging, which is consistent with the characteristics of the ontological health concept [39], which is perceived as a movement toward wholeness and holiness [40]. The four descriptive categories are consistent with the three health levels in the ontological health concept: health as behavior, health as being, and health as becoming [39]. Herberts and Eriksson [39] described the first dimension of health as having health, which is a way of achieving health through certain behaviors that are considered healthy by the individual or society. The conceptions of healthy aging as being independent and not being afflicted by diseases or illnesses are related to the first dimension of health, that is, having health. Healthy aging also entails being independent, having the ability to care for oneself, being free from diseases or illnesses, and maintaining strong bodily and cognitive functions. Healthy aging is consistent with the idea of evaluating health using external objective criteria and focusing on illness-related problems [39].

A comparison of the meaning of healthy aging found in this study and that reported in other studies reveals explicit differences and similarities. Regarding the description of the nature of each category, the conceptions of healthy aging as being independent and not afflicted by diseases or illnesses are consistent with the ideas expressed by healthcare professionals in earlier studies of healthy aging in Western countries [5, 6]. We observed that being independent was a criterion for healthy aging, which may indicate that Thai cultural values have been influenced by Western traditions, in which individualism plays a prominent role [24, 41]. Thus, modern society may have an impact on people's beliefs and normative contexts. A previous study showed that, according to the perspective of older persons in the Isan region, healthy aging still depends on one's relatives [7]. Furthermore, older persons are dependent on caregivers [21, 42], whereas their younger relatives may value independence. While older persons still need assistance [21] because of changes in their bodily functions [43], the children and grandchildren of older persons conceptualize healthy aging with respect to the functional capacity of older persons and not being a burden on relatives. These conceptions may be partly related to ageism, the belief that older persons are inferior to younger people because of their age [44]. Children and grandchildren view older persons as being different from themselves, which may result in negative attitudes toward older persons. This study indicates that family nurses should be aware of these conceptions when collaborating with the children and grandchildren of older persons to promote healthy aging. Considering these aspects, policies should be developed according to the conception of healthy aging with respect to the burden of older persons on family members and the roles of older persons in society. Another suggestion is to support older persons when they lack the capacity to increase the value of their lives. Policy-makers and family nurses should emphasize the value of older persons to younger people.

Healthy aging is also defined as “being a giver and a receiver.” This category is related to the second dimension of the ontological health concept, in which health is viewed as “being health” [39]. Health is characterized as the achievement of balance and harmony, whether between the body and soul; between physiological, psychological, and social factors; or between internal and external elements [39, 45]. When comparing that conception of healthy aging to the meaning found in this study, there are differences between the categories “being independent” and “being a giver and a receiver.” Surprisingly, “being independent” and “being a giver and a receiver” seem to be contradictory. The meaning of healthy aging that focuses on healthy aging as a state characterized by independence may reflect the influence of the Western perspective [5]. In contrast, “being a giver and a receiver” stresses the importance of the relationships between older persons and their family members as an influential factor in healthy aging, which may be due to the values of Isan culture. Being a giver and a receiver is related to the dynamics of the relationships between children and grandchildren and older persons in Isan culture. This relationship reflects the idea that children are indebted to their parents because their parents raised them from birth [22, 42]. Thus, these relationships may be related to the concept of intersubjectivity, which is defined in phenomenological theory [29, 46, 47] as the idea that everyone relies on and exists in relation to others [48]. The children and grandchildren of older persons interact with older persons throughout their lives and use their own bodies to understand how the concept of healthy aging relates to the older persons with whom they live. Parents provide for their children, love them, and take care of them from early childhood, and children are taught that they should live with and repay their parents until the parents die [22]. This connection may be described as an obligation and a social norm in Isan-Thai culture, which is based on Buddhism. Although the Isan lifestyle has been changed by modernization [24], people in this region continue to have a strong belief in Buddhism [49]. Isan customs and Buddhist beliefs are passed down from generation to generation [50], and the cultural values tied to these customs and beliefs determine the individual person's thoughts and ideas [51]. Isan people cling to their culture through local customs [49]. Buddhists believe in the concept of karma [22, 52], which means that one receives what he or she gives to others; this means that whatever one does to others may also be done to him/herself in the future. This belief is illustrated by the practice of making merit, which, according to Buddha's teachings, brings happiness and peace [9]. Isan values are rooted in the interactions between an individual and others. Thus, this category is related to the interdependence [8, 53] that characterizes Isan society. This finding indicates that family nurses should endeavor to enhance the relationships between older people and their children and grandchildren to promote healthy aging.

In the final category, healthy aging is conceived of as “being wise.” Comparisons between our results and those of previous studies regarding healthy aging [5, 6] indicate that the domains of one's experience, adaptation, and preparation are related to wisdom. Being wise is related to the last dimension of health, in which health is perceived as “becoming” [39], meaning that human beings are becoming something greater than themselves and/or becoming whole at a deeper level [39]. In this context, wholeness refers to physical, mental, and spiritual wholeness [39]. Furthermore, Eriksson [54] noted that health as becoming means that “the person strives to reconcile himself with the circumstances of life and to become whole in a deeper dimension of integration” (p. 76). Our findings indicate that being wise is linked to self-adjustment and to one's ability to face his or her problems and the changes associated with the aging process. People in society believe that older persons have gained important life experiences that enable them to face life's problems and thus consider these life experiences as valuable sources of wisdom [55]. Wisdom is believed to benefit older persons in facing life situations and is correlated with older age [56, 57]. Wisdom also underlies the respect shown to older persons by children and other relatives who greatly admire them [58]. Another explanation for this behavior is consistent with the idea of ego integrity [59, 60]. This category may be associated with the ability to integrate one's life experiences. This finding suggests that the life experiences and wisdom of older persons should be revealed in order to promote positive attitudes toward older persons. We observed that participants also defined healthy aging as preparation for aging.

The findings of this study represent a starting point for family nurses and/or policy-makers when considering the perspectives of young adults regarding healthy aging. For the future challenges of supporting healthy aging, we suggest that family nurses plan for the promotion of healthy aging with adults in parallel with the current older population, according to the findings of this study. For example, family nurses should serve the workforce and programs for preparing healthy aging in middle adulthood. These programs should be related to the prevention of health problems. Moreover, the promotion of healthy aging should be relevant to the different conceptions of healthy aging. The family members of older persons should be encouraged to brainstorm a list of the key determinant factors in supporting their health and advance care planning to promote healthy aging.

### 4.1. Methodological Considerations

Rigor in phenomenographic research is based on the study's validity and reliability [61]. The participants of this study were carefully selected using a strategy intended to obtain a varied and broad picture regarding the focus of the study—the conceptions of healthy aging held by the relatives of older persons. The number of participants recruited for this study was considered reasonable, but the participants included more women than men. Thus, the conceptions of healthy aging held by the men who participated in this study may not be fully representative. In-depth interviews exploring the conceptions of healthy aging were conducted by one person (the first author), which allowed probing for deeper elaboration and ensuring that all information was considered. Specific quotations were used to clarify the meaning of healthy aging. The rigorous approach used in this study also yielded findings that are transferable [62]. This study focused on the conceptions of healthy aging held only by the children and grandchildren of older persons who live in the Isan region of Thailand. This restriction may have influenced our findings, as cultural backgrounds may impact people's beliefs. However, the findings of this study may be used to transfer to the relatives of older persons with similar backgrounds, such as the children and grandchildren of older persons who live in the northeast of Thailand. Although the results of the present study can be related to the Western perspective on the meaning of healthy aging, data were drawn from the relatives of older persons in only one region of Thailand. The information about the meaning of healthy aging gathered from this group of family members of older persons cannot be applied to the entire world. The purposive sampling method may also limit the generalizability of our results. Moreover, all the participants were Buddhists. Although Buddhism is a main religion in Thailand, the conceptions of healthy aging held by Buddhists may differ from those held by members of other religions.

## 5. Conclusions

The key findings of this study are the conceptions of healthy aging represented as a set of categories: “being independent,” “not being afflicted by diseases or illnesses,” “being a giver and a receiver,” and “being wise.” Furthermore, the meaning of healthy aging is linked to older persons, their relatives, and the Buddhist religion, which emphasizes the importance of a holistic view of healthy aging. Although some relatives' conceptions of healthy aging were consistent with Western perspectives, traditional Isan-Thai cultural values still influence the meaning of healthy aging. This study highlights the need to be aware of the unique conceptions held by the children and grandchildren of older persons in the Isan region when promoting healthy aging.

## Figures and Tables

**Table 1:** Participants' characteristics (*n* = 14).

Number	Sex	Age	Relation with older person	Education	Marital status	Occupation	Religion
1	F	16	Granddaughter	High school	Single	Student	Buddhism
2	F	42	Daughter	Elementary	Married	Housewife	Buddhism
3	F	45	Daughter	Elementary	Married	Farmer	Buddhism
4	F	34	Daughter	High school	Married	Housewife	Buddhism
5	M	41	Son	High school	Married	Employee	Buddhism
6	M	24	Grandson	High school	Single	Self-employed	Buddhism
7	M	46	Son	High school	Single	Self-employed	Buddhism
8	F	41	Daughter	Master degree	Single	Teacher	Buddhism
9	F	56	Daughter	Bachelor degree	Married	Teacher	Buddhism
10	F	40	Daughter	Master degree	Single	Teacher	Buddhism
11	F	54	Daughter	High school	Single	Employee	Buddhism
12	F	48	Daughter	Elementary	Married	Self-employed	Buddhism
13	F	40	Daughter	Elementary	Married	Farmer	Buddhism
14	F	42	Daughter	Elementary	Married	Employee	Buddhism
